# Next-generation sequencing for cytomegalovirus genotypic antiviral resistance testing

**DOI:** 10.1128/jcm.01302-23

**Published:** 2023-11-22

**Authors:** Heba H. Mostafa

**Affiliations:** 1 Department of Pathology, Division of Medical Microbiology, Johns Hopkins School of Medicine, Baltimore, Maryland, USA; Johns Hopkins University, Batimore, Maryland, USA

## Abstract

Sanger-based sequencing has long served as the gold standard for detecting cytomegalovirus (CMV) resistance mutations. However, next-generation sequencing (NGS) offers a highly multiplexed and sensitive approach. Clinical implementation of NGS-antiviral resistance testing requires thorough validation. In this issue of the Journal of Clinical Microbiology, M. A. Mallory, W. C. Hymas, K. E. Simmon, M. T. Pyne, et al. (J Clin Microbiol 61:e00829-23, 2023, https://doi.org/10.1128/jcm.00829-23) detail a validation of a targeted NGS-based assay for multiple genomic regions that confer resistance to CMV antivirals and share their bioinformatics analysis and reporting pipeline. This validation can serve as guidance for laboratories wishing to develop similar methodologies.

## COMMENTARY

The genotypic characterization of resistance mutations has become the test of choice for characterizing antiviral resistance. For human cytomegalovirus (CMV), genotypic resistance testing is recommended when treatment failure is a concern (1 – 3). Currently, six FDA-approved drugs are used for managing CMV infections: ganciclovir (a guanosine nucleoside analog, viral UL97 kinase activated), valganciclovir (a prodrug of ganciclovir), cidofovir (a cytidine monophosphate analog), foscarnet (a viral UL54 polymerase inhibitor), maribavir (a viral UL97 kinase inhibitor), and letermovir (a viral terminase complex inhibitor) (4). Studying the genetic mutations associated with CMV resistance to these drugs has further characterized their mechanisms of action. Mutations conferring resistance to ganciclovir and maribavir in *ul97* (encoding for UL97 kinase), cidofovir, foscarnet, and ganciclovir in *ul54* (encoding for UL54 DNA polymerase), letermovir in *ul56, ul89,* and *ul51* (encoding for subunits of the terminase complex), and maribavir in *ul27* have been mapped. Resistance genetic loci continue to expand, and novel resistance mutations are continuously being identified.

Sanger-based sequencing has long been the gold standard for genotypic CMV resistance testing, primarily targeting codons 440–670 for UL97 and 300–1000 for UL54 (5, 6). Given the newly approved drugs and identified resistance mutations, these genes and codons are not fully comprehensive. Expanding the sequencing regions has become clinically warranted, and assays capable of multiplexing gene targets are preferable. The Sanger-based techniques available in large reference laboratories necessitate bidirectional amplification of multiple amplicons, which, at present, do not encompass all clinically significant resistance mutations. While Sanger sequencing can be scaled up and offers ease of standardization across diverse laboratories, the use of multiple amplicons can result in higher associated costs. Overall, the limitations of Sanger-sequencing approaches include (i) sequencing one gene target per reaction, increasing the test cost, (ii) low sensitivity for low-frequency and minor variants, with a threshold of variant detection of ≥20%, and (iii) reduced quality for sequences beyond 700 base pairs (6, 7).

Previous studies have characterized the performance of next-generation sequencing (NGS) for CMV resistance testing and emphasized its increased sensitivity and improved detection of low-frequency mutations (8 – 11). NGS-based approaches offer several advantages including: (i) improved analytical performance by increasing sensitivity for identifying minor variants and (ii) enhanced efficiency in the clinical laboratory and cost-effectiveness by multiplexing targets and samples. Both Sanger- and NGS-based methods offer flexibility for expanding sequencing regions and targets to cover all approved antivirals. Nevertheless, given the significant advancements in NGS technologies, the clinical viability of these robust methods, along with their current adaptability to clinical settings, justifies the growing interest in incorporating NGS into routine clinical care. Reference laboratories, such as Mayo Clinic and ARUP, currently offer CMV resistance testing using NGS. The ARUP NGS assay detects mutations in UL27, UL97, UL54, and UL56 and is currently more clinically comprehensive. This assay has been in relatively high demand by institutions that frequently prescribe maribavir and letermovir for their patient populations.

Mallory et al. (12) report the clinical validation of an ARUP NGS-based targeted assay for detecting mutations in *ul27*, *ul54*, *ul56*, and *ul97*. They compare the results to Sanger-based standard of care and research-use-only assays. Their NGS assay spans a 10-KB genomic region containing 138 resistance amino acid substitutions and deletions. Notably, the authors’ NGS assay multiplexes the sequencing of 83 overlapping amplicons in each reaction. In contrast, the clinical Sanger-based assay bidirectionally sequences one amplicon spanning UL97 (codons 457–650) and uses four overlapping amplicons for UL54 (codons 393–1000). Therefore, the NGS assay expands the sequencing regions to be more inclusive of additional resistance mutations in a highly multiplexed fashion.

A major challenge in interpreting NGS results is the exclusion of sequencing artifacts, especially when the target load is low or when characterizing minor variants is desirable. Mallory et al. (12) conducted a thorough analytical evaluation of the NGS assay’s sensitivity and reproducibility. The authors designed and generated plasmids containing wild-type or mutant sequences for *ul27*, *ul54*, *ul56*, and *ul97*. They used these plasmids to assess the NGS assay’s error rate and its sensitivity for detecting minor variants when wild-type and mutant plasmids were mixed in different ratios. Reproducibility and error analysis showed that the majority of sequencing errors occur at low copy numbers when they are present at <5% frequency. Therefore, a threshold of 10% was selected as the optimal cutoff for calling true positives. A clinical validation that compared the NGS results of 185 patients to Sanger-generated sequences was conducted. Plasma specimens, which had previously been tested using the ARUP Sanger-based CMV resistance test as the standard of care, were retested using the NGS method. A subset of samples (30) was also subjected to testing for resistance mutations outside of the genomic areas covered by the standard of care Sanger assay, utilizing a research-based Sanger-sequencing method. Overall, there was a 98.6% agreement between the NGS- and Sanger-based methods. Importantly, NGS identified resistance mutations in 16 samples that were not detected by the Sanger-based method, and 6 of these mutations would have led to a different reporting interpretation. Taken together, the NGS approach showed enhanced sensitivity and high reproducibility at a lower cutoff threshold than Sanger sequencing.

A significant challenge in applying NGS for clinical testing is standardization, primarily due to differences in assay design, sequencing platforms, and, most importantly, analysis pipelines. CMV resistance testing by NGS has been evaluated using various sequencing chemistries, including Ion Torrent, Illumina, and Oxford Nanopore Technologies, and resistance mutation analyses were performed using different bioinformatics pipelines (8 – 11). Quality metrics and thresholds for calling resistance mutations can contribute to sequencing artifacts and false positives, and the quality of data and the rigor of bioinformatics analysis are key factors in ensuring a high-quality NGS-based diagnostic method. Mallory et al. (12) publicly shared their algorithm for calling resistance mutations and reporting results, along with a user guide and a resistance mutations database, to be used for sequences generated with compatible Ion Torrent platforms. Their data analysis incorporated strict quality cutoffs, with a minimum read depth of 40× for any of the 138 resistance mutation codons being required to distinguish between conclusive and indeterminate results. This resource sharing will undoubtedly facilitate standardization and assay development across different laboratories.

In conclusion, given the burden of CMV infections and their associated poor outcomes in immunocompromised patients, it is crucial to rapidly identify mutations associated with antiviral resistance and understand their clinical implications. The analytical sensitivity of NGS is reportedly consistently higher than that of Sanger-based methods, which can have a significant clinical utility (11). NGS is currently the most sensitive and inclusive option; however, due to the high complexity, sequencing-based assays are currently primarily offered by reference laboratories, and thorough analytical and clinical validations are essential before their clinical implementation. Automation, coupled with user-friendly analysis solutions, can increase the testing capacity and reduce the time to obtain results, thereby enhancing the clinical utility of such assays. Whole-genome sequencing may be the future approach for quickly identifying novel mutations, and this can only be accomplished by adopting NGS-based methods.
